# A Case of Resection of Hepatocellular Carcinoma in a Patient With Fontan-Associated Liver Disease

**DOI:** 10.7759/cureus.33382

**Published:** 2023-01-05

**Authors:** Satoshi Tokuda, Hideyuki Kanemoto, Akihiko Takagi, Yuichi Masui, Noriyuki Oba

**Affiliations:** 1 Department of Gastroenterological Surgery, Shizuoka General Hospital, Shizuoka, JPN; 2 Department of Gastroenterology, Shizuoka General Hospital, Shizuoka, JPN

**Keywords:** hepatectomy, cvp, fald, hepatocellular carcinoma, fontan procedure

## Abstract

The Fontan procedure (FP) is an operation used in patients with congenital single ventricle disease. The long-term prognosis after surgery has improved due to technological advances. However, the hemodynamics after FP are complicated. There are some reports of Fontan-associated liver disease (FALD) after FP. We report a case of a young woman who developed hepatocellular carcinoma due to FALD.

## Introduction

The Fontan procedure (FP) is an operation used in patients with congenital single ventricle disease. The procedure involves anastomosis of the superior and inferior vena cava directly to the pulmonary artery without going through the right ventricle. Elevation of central venous pressure (CVP) is necessary to promote pulmonary circulation in FP [1]. The long-term prognosis after the surgery has improved due to technological advances [2]. However, the hemodynamics after FP are complicated, and there are some reports of liver disease after the Fontan procedure, particularly Fontan-associated liver disease (FALD). In the patients, the hepatic veins are more susceptible to central venous influences because they are anastomosed directly into the Fontan circulation. Increased CVP and decreased cardiac output can lead to reduced portal vein inflow. In the Fontan circulation, decreased portal blood flow and oxygen saturation in the portal vein are expected, and the liver will suffer ischemic damage. Chronic ischemic damage to the liver can cause FALD, which can lead to liver fibrosis, cirrhosis, and hepatocellular carcinoma [3]. CVP is a key factor in FP. Here, we report a case of a young woman who developed hepatocellular carcinoma (HCC) due to FALD and underwent surgery.

## Case presentation

**Figure 1:**
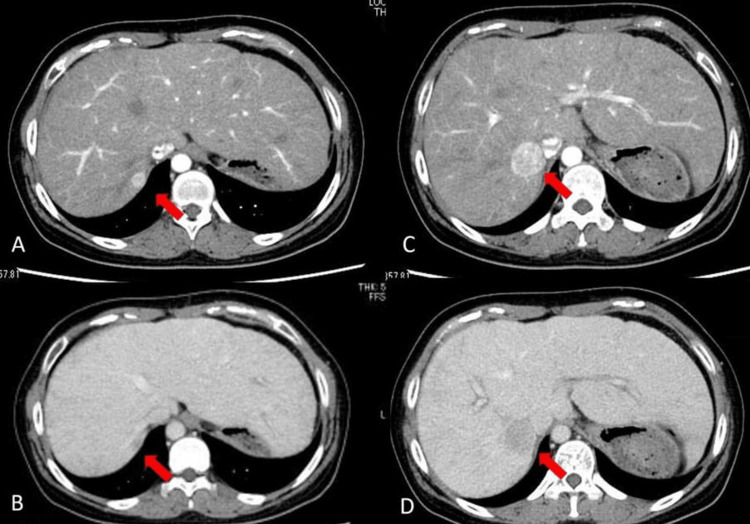
Hypervascular masses in segment VII on arterial phase, followed by delayed washout (A) Arterial phase, (B) Delayed phase, (C) Arterial phase, (D) Delayed phase A and B & C and D are the same lesions, respectively

In a 27-year-old female patient, cyanosis had been recognized at birth. It was pointed out again at the four-month checkup. A thorough examination revealed dextrocardia, congenital asplenia syndrome, single atrium, single ventricle, and pulmonary atresia. She had undergone the Glenn procedure (the operation connects the superior vena cava to the pulmonary artery) and pulmonary angioplasty at the age of two years for single ventricle and pulmonary atresia. At the age of seven years, she had undergone FP and developed cirrhosis due to liver damage from FALD. She was regularly monitored postoperatively. A routine follow-up CT scan revealed liver masses. Serum levels of the tumor markers alpha-fetoprotein (AFP) and protein induced by vitamin K antagonists-II (PIVKA-Ⅱ) were elevated to 270 ng/ml (normal < 10 ng/ml) and 415 mAU/ml (normal < 40 mAU/ml), respectively. Additional laboratory test results were normal, except for elevated alanine transaminase levels of 42 U/l (normal, 7-23 U/l). Tests for hepatitis B and C viruses were negative. She had no history of heavy alcohol drinking. Indocyanine green retention rate was 3.5% at 15 min. Values of type IV collagen and hyaluronic acid, which are markers of liver fibrosis, were 4.9 ng/ml (normal < 4.4 ng/ml) and 43 ng/ml (normal < 50 ng/ml), respectively. Liver function tests were normal with Child’s A cirrhosis. The patient weighed 51.5 kg, with a height of 140 cm. Oxygen saturation on room air was approximately 97%. Echocardiogram revealed a left ventricular ejection function of 55%, and electrocardiography displayed normal sinus rhythm. Cardiac catheter test and cardiopulmonary exercise testing, which were routinely performed three months before the operation, revealed a CVP of 14 mmHg, cardiac output of 2.50 l/min, left ventricular ejection function of 56.0%, and peak VO2 (oxygen consumption) of 22.9 ml/kg/min (66% of standard value), VO2 at AT trend of 12.2 ml/kg/min (77% of standard value), VO2 at AT V-slope of 12.2 ml/kg/min (78% of standard value), min VE/VCO2 of 34.1 (122% of standard value), VE/VCO2 slope of 20.9, ΔVO2/LOAD of 6.07 (59% of standard value), and peak VO2/HR of 5.8 (82% of standard value). Based on the above test results, the patient was examined at the department of congenital heart disease before surgery and was deemed operable. She had two hepatic lesions in segment Ⅶ, measuring approximately 25 mm and 10 mm, respectively, on CT scan arterial enhancement and portal venous washout (Figure 1). 

**Figure 2:**
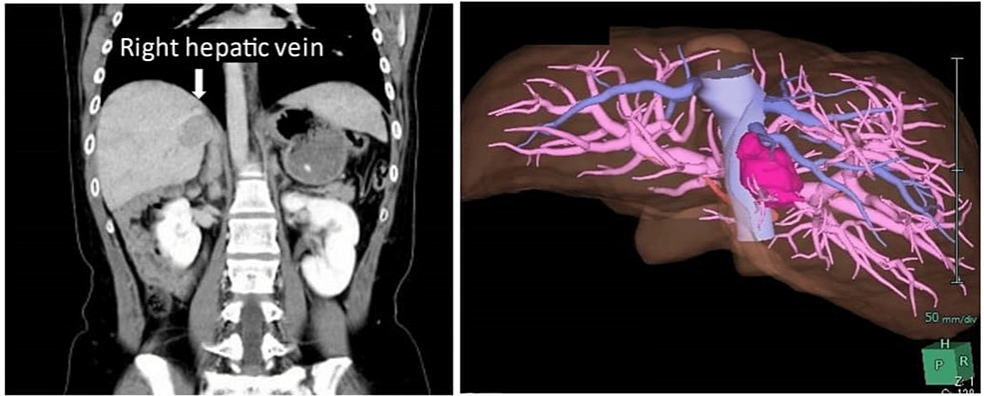
The tumor location is adjacent to the right hepatic vein

HCC was diagnosed. CT revealed no ascites or collateral circulation. The tumors were close to the right hepatic vein (Figure 2) and extended right posterior sectionectomy was selected as the surgical technique. The remnant liver volume was estimated at 1213 ml (74.9%). There were two reasons for selecting this operative method: prevention of congestion of segment Ⅵ and location of the tumors. The tumors were very close to the right hepatic vein, increasing the risk of injuring the blood vessel and subsequent difficulty to control the resulting bleeding. Aspirin intake was stopped seven days prior to surgery. An autologous blood sample was taken before the surgery.

Surgical procedure

During surgery, an arterial line and a central venous catheter were inserted to accurately monitor the hemodynamics of the patient. We opted for an inverted L-shaped incision. The intraoperative ultrasound of the liver revealed two lesions in segment Ⅶ, with the larger lesion located close to the right hepatic vein. We performed extended posterior sectionectomy as scheduled. Surface roughness with congestive liver was observed and considered to be cirrhosis. The hepatoduodenal ligament was taped for the Pringle maneuver. Furthermore, we mobilized the right lobe of the liver and cut the right adrenal vein using an automatic suture device. The mobilization was not so difficult. The Pringle maneuver was performed to control operative blood loss in liver dissection; however, we did not use inferior vena cava (IVC) clamping to prevent low blood pressure. Cavitron ultrasonic surgical aspirator (CUSA) was initially used for hepatic parenchymal dissection but due to the hardness of the tissue, a clamp crush method was used. Fine vascular vessels were processed with ultrasonic transection and coagulation systems. We did not perform the hanging maneuver. Exudation from the hepatic incision surface was noted but not uncontrollable. After the preceding hepatic transection, Glisson's branches of segment 8c and the posterior section were processed. Finally, the right hepatic vein was dissected and the specimen was removed. A drain was placed on the liver dissection surface. During the Pringle maneuver, the mean CVP was 11 mmHg, and it was 14 mmHg during the declamping. No uncontrolled hypotension was observed. The duration of the operation was 4 h 40 min, and the amount of bleeding was 650 ml.

Histological findings

**Figure 3:**
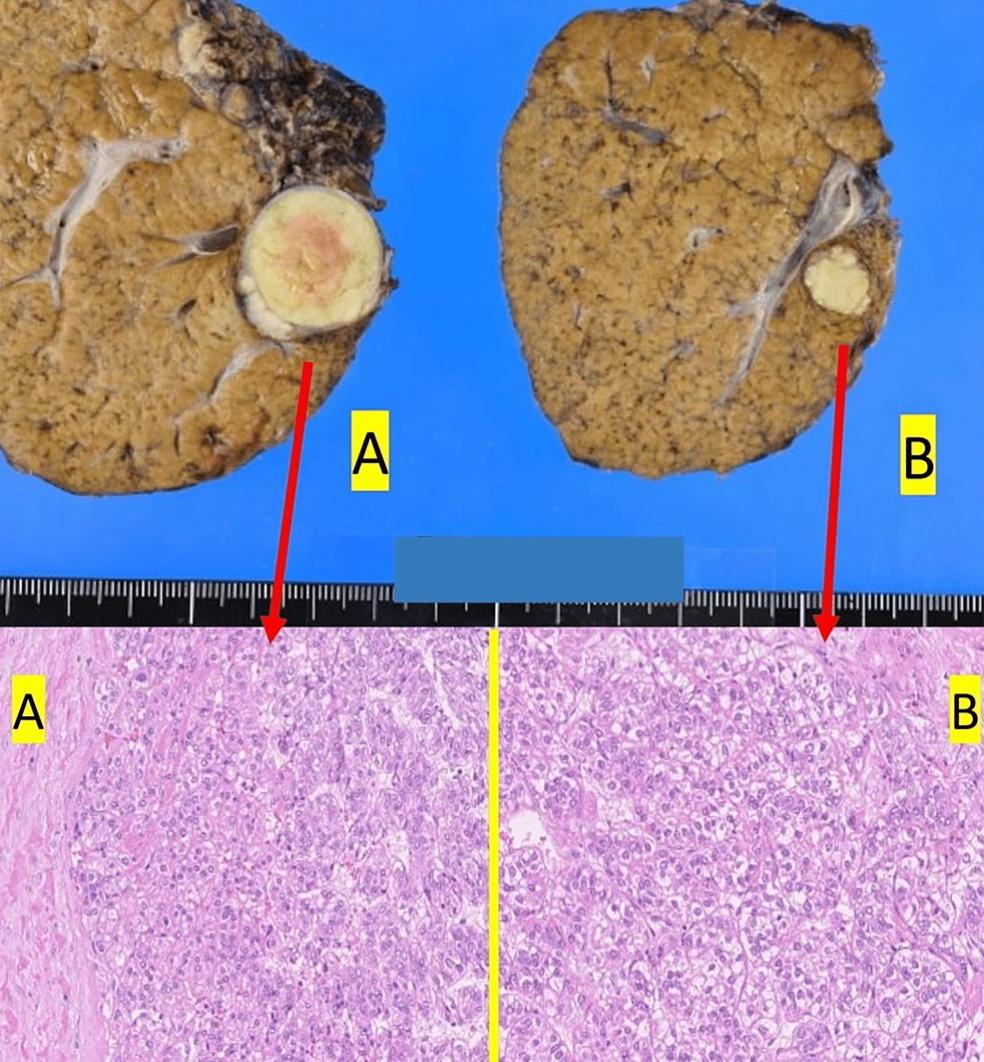
The background liver is A1F3, and the tumors are moderately differentiated HCC, respectively (A) 2.8×2.3 cm, simple nodular type, Fc(+), Fc-inf(-), Sf(+), sm(-, 0.1 mm) (B) 1.2× 0.9 cm, simple nodular type, Fc(-), Fc-inf(+), Sf(-), sm(-, 37 mm)

The tumor findings were as follows: (A) moderately differentiated HCC, 2.8×2.3 cm, SM (-, 0.1 mm) and (B) moderately differentiated HCC, 1.2×0.9 cm, SM (-, 37 mm). The peritumoral liver tissue showed stage F3 cirrhosis according to the new Inuyama classification (Figure 3). 

Pathological findings showed no tumor invasion into the right hepatic vein.

Postoperative course

The patient was managed in the high-care unit for three days after surgery. The target CVP was 10-15 mmHg, and diuretics were administered as appropriate, paying attention to the volume load. The number of diuretics was adjusted by a cardiologist (Table 1).

**Table 1:** Amount of diuretics adjusted by a cardiologist iv: intravenous injection; p.o.: per os; POD: postoperative day

POD	Furosemide	Soldactone®
1	60 mg/day iv	200 mg/day iv
2	60 mg/day p.o.	75 mg/day p.o.
4	30 mg/day p.o.	75 mg/day p.o.
6	20 mg/day p.o.	75 mg/day p.o.

**Figure 4:**
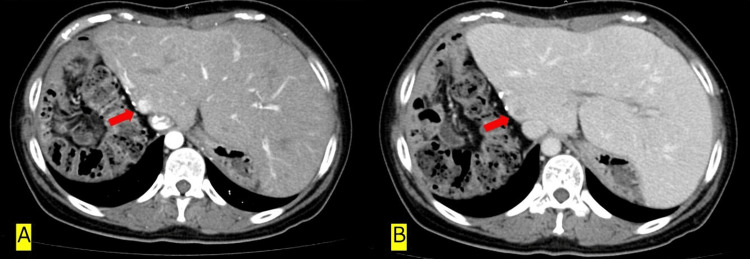
Recurrent tumor of S1 (A) Arterial phase, (B) Delayed phase

**Figure 5:**
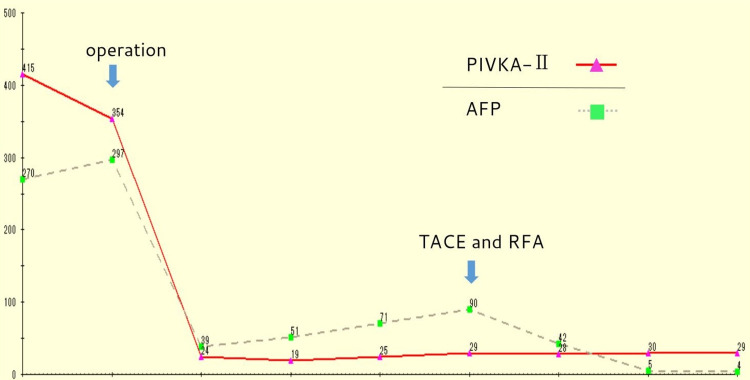
Tumor marker trends

The amount of drain discharge was in small amounts after the operation. The patient had no major side effects and was discharged eight days after surgery. Furosemide and Soldactone were used together for the first three months postoperatively, after which Soldactone alone is continued. At seven months after surgery, HCC recurrence was detected in segment 1 (Figure 4) and the patient underwent transarterial chemoembolization (TACE) and radiofrequency ablation (RFA). Tumor marker trends are shown below (Figure 5).

No new lesions were observed at 21 months postoperatively.

## Discussion

As mentioned above, there have been postoperative reports of HCC in patients who have undergone FP. However, there are few reports of actual hepatic resection due to liver cirrhosis and poor cardiac functional reserve [4]. Studies pertaining to HCC with FALD on PubMed are presented in Table 2 [1,4-10].

**Table 2:** Studies on HCC patients with FALD in PubMed FALD: Fontan-associated liver disease; HCC: hepatocellular carcinoma

Author	age	sex	height(cm)	weight(kg)	chief complaints	tumor location	size (cm, max)	operative method	operation time(min)	blood loss(ml)	complication	postoperative hospital stay	outcome
Paul, 2014 [5]	23	female	164	56	abdominal pain and mass	right lobe	14.8	right hepatectomy	ND	1000	no complication	ND	ND
Steve, 2015 [1]	32	male	ND	ND	routine follow-up	S7	4	partial right hepatectomy	ND	1500	fluid overload, pleural effusions	9	12 months alive
Yoshitaka, 2016 [6]	29	female	ND	ND	abdominal discomfort	S4	1.5	partial hepatectomy	ND	ND	no complication	ND	12 months alive
Dorsey, 2016 [7]	32	male	ND	68	routine follow-up	S7	4	S7 segmentectomy	ND	1500	pleural effusions	9	6 months alive
Kevin, 2018 [8]	24	female	ND	ND	routine follow-up	left lobe	10.8	left hepatectomy	510	4100	no complication	12	6 months alive
Angelico, 2019 [9]	33	female	ND	ND	tumor marker elevation	S5	4.5	laparoscopic wedge resection	ND	100	no complication	7	7 months alive
Yokota et al., 2020 [10]	18	male	ND	ND	routine follow-up	S2 and S4	ND	laparoscopic wedge resection	327	20	no complication	9	12 months alive
Nemoto, 2020 [4]	37	female	157	43	epigastralgia	caudate lobe	8	Left hepatectomy and caudate lobectomy	450	3200	ascites	9	16 months alive
Iwata, 2021 [11]	31	male	ND	ND	routine follow-up	S3	1.5	laparoscopic partial liver resection	117	10	no complication	3	7 months alive
Present case	27	female	140	51.5	routine follow-up	S7	2.5	post-enlargement segmental resection	280	650	no complication	8	21 months alive
ND (not described)													

The mean duration of FP to a diagnosis of cirrhosis was 23.4 years [3]; accordingly, the age of onset of HCC has also decreased. Depending on the operative method, the amount of intraoperative blood loss seems to be relatively high; however, laparoscopic surgery is also possible if the appropriate case is selected. In the present case, major hepatectomy could be performed safely with relatively little blood loss by referring to previous reports on the procedure. Concerning intraoperative management, while control of blood loss is important, the Pringle maneuver does not have adverse effects on the Fontan circulation [10]. However, IVC clamping is prone to hypotension in the Fontan circulation [4]. The reverse Trendelenburg position is a safer technique, without decreasing blood pressure, than IVC clamping [12]. In the present case, the Pringle maneuver, without IVC clamping and reverse Trendelenburg position, was effective in controlling blood loss, but other methods should be considered, depending on the situation. Anesthesiologists need to pay attention to CVP and manage it accordingly to maintain circulation. In perioperative management, peripheral vascular resistance is expected to decrease due to inflammation after hepatectomy. Excessive infusion restriction leads to circulatory compromise. However, pulmonary congestion from volume overload results in a significant increase in both pulmonary vascular resistance and CVP, which leads to circulatory compromise and organ congestion. It is important that cardiologists cooperate due to the narrow safety zone.

## Conclusions

Careful preoperative tolerance testing is necessary when performing hepatic resection in patients who have undergone FP. They should be managed perioperatively in cooperation with anesthesiologists and cardiologists, using intraoperative and postoperative CVP as one indicator. In addition to that, we surgeons should also devise ways to reduce intraoperative blood loss. For the above reasons, surgery at a high-volume center is preferred for such patients.
